# Avoidance behaviours of farmed Atlantic salmon (*Salmo salar* L.) to artificial sound and light: a case study of net-pen mariculture in Norway

**DOI:** 10.3389/frobt.2025.1657567

**Published:** 2025-09-11

**Authors:** Qin Zhang, Nina Bloecher, Linn Danielsen Evjemo, Martin Føre, Eleni Kelasidi

**Affiliations:** 1 Department of Engineering Cybernetics, Norwegian University of Science and Technology, Trondheim, Norway; 2 Department of Aquaculture, SINTEF Ocean AS, Trondheim, Norway; 3 Department of Mechanical and Industrial Engineering, Norwegian University of Science and Technology, Trondheim, Norway

**Keywords:** aquaculture, *Salmo salar*, behaviour, deep learning, robotics and automation, fish farm design inputs

## Abstract

Intensive finfish aquaculture is increasingly relying on enabling technologies and solutions such as sensor systems, robotics, and other machinery. Together with conventional farming equipment, these systems may emanate acoustic noise and artificial light, impacting the pen environment. Farmed fish have been observed to respond behaviourally and/or physiologically to anthropogenic sounds and lights, indicating a stress reaction that could impair welfare and health. This study aimed to investigate how farmed Atlantic salmon respond to such stimuli, with direct implications for the design and operation of robotic and mechanised systems in sea pens. We conducted experiments where we systematically exposed adult farmed Atlantic salmon in commercial net pens to sounds of frequencies within the range common to farm equipment (100–1,000 Hz), and submerged lights at 8 and 12 m with four different intensities (600 lx–14,500 lx). Data was analysed using sonar data and a deep learning (DL) based method for processing that automatically identified fish distribution patterns and estimated the average avoidance distance to the sound/light source. The fish fled from the sound source while playing sounds of 400 Hz, while sounds at other frequencies did not elicit a response. The response to light intensity depended on deployment depth, with the fish moving closer to the source when intensity was increased at 8 m depth, but conversely moving further away with increasing density when it was placed at 12 m. These outcomes are important inputs for the design of equipment, autonomous vehicles, robotic interventions and operations at commercial farms to ensure that their sound and light emissions have minimal impact on the fish, thereby reducing the potential of induced stress.

## 1 Introduction

Norwegian aquaculture is a rapidly growing industry that is experiencing a shift from a regime of manual operation and experience-based reasoning to knowledge-based approaches that integrate smart sensors, mathematical models, decision support systems and autonomous methods (Føre et al., 2018). In addition to exploring more exposed and remote locations, the industry is generally increasing the size of individual production sites (Bjelland et al., 2015; 2024), further contributing to making fish farm operations more dependent on technology and automation. At the same time, focus on fish welfare is constantly increasing (Sommerset et al., 2024) with the aim to create an ideal rearing environment where stress from infrastructure or operations is minimised.

One of the aspects that has received more attention lately is the impact of acoustic noise which is assumed to be an important factor in both tank and pen based intensive aquaculture (Radford and Slater, 2019; Barrett and Oppedal, 2025). The general soundscape at sea farms is generated by the combined impacts of a plethora of different sources (Oppedal et al., 2024; Barrett and Oppedal, 2025). While some of the sounds are generated by the fish and their activities (Rosten et al., 2023), most are caused by man-made systems and activities (Radford and Slater, 2019; Popper et al., 2003). This includes, for example, service vessels (Barrett and Oppedal, 2025) but also mobile equipment used inside the pens, such as Remotely Operated Vehicles (ROVs) Kelasidi and Svendsen (2023), that use propellers or water jets (Cai and Bingham, 2011). In addition, constantly running farm equipment (Wysocki et al., 2007) such as feed blowers will also add to the acoustic noise, as will general human presence and activities at the farm. While this implies a complex acoustic picture, it is currently unclear how much of this noise is perceived by the fish, and how they react to it.

Atlantic salmon have a hearing range of about 100–380 Hz (Hawkins and Johnstone, 1978; Oxman et al., 2007), with laboratory experiments showing reactions up to 580 Hz (Hawkins and Johnstone, 1978). However, results from Oxman et al. (2007) and Reimer et al. (2016) showed that farmed salmon tend to have significantly impaired hearing compared with wild salmon due to otolith deformities. This may impact how they perceive the acoustic environment, and hence their propensity to respond towards acoustic noise. Nevertheless, previous studies have found sounds able to trigger stress responses in fish, often manifested as an increase in levels of stress hormones such as cortisol (Sierra-Flores et al., 2015; Wysocki et al., 2006; 2007; Oppedal et al., 2025). Others have observed that long-term exposure to high intensity noise can cause fish to experience ongoing physiological stress, which in turn may affect their immune systems and make them more susceptible to disease (Quinn, 2007; Masud et al., 2020; Sholes and Coffin, 2023). In terms of auditory impacts, repeated or prolonged exposure to loud sounds has been shown to cause temporary hearing loss, and recovery times range from days to weeks, depending on species and exposure levels (Popper and Clarke, 1976; Scholik and Yan, 2002; Smith et al., 2004). For example, Smith et al. (2004) found that goldfish took more than 2 weeks to fully recover after 3 weeks of exposure to moderate-intensity sound (170 dB re 1 µPa), while Scholik and Yan (2001) found that fathead minnows failed to recover to control hearing levels even 14 days after 24-h noise exposure.

Sound exposure may also affect the behaviour patterns of fish since many fish species actively use sounds for communication, foraging, avoiding predators and reproduction, as described in detail for aquarium fish such as gobies (De Jong et al., 2018). While farmed fish display a more limited set of behaviours than wild fish, acoustic noise has been found to disturb their swimming patterns (Hang et al., 2021). Studies have shown significantly reduced foraging behaviour in fish when exposed to vessel noise (Magnhagen et al., 2017; Pieniazek et al., 2020). Pieniazek et al. (2020) reported that fish with more sensitive hearing exhibited a greater reduction in feeding activity under boat noise exposure. Similarly, Magnhagen et al. (2017) found that motorboat noise significantly impaired foraging, reinforcing concerns that anthropogenic noise interferes with essential behaviours. Fish may also show escape responses to sounds. Oppedal et al. (2025) observed salmon reacting to high power low frequency sounds (10 Hz) with flight behaviour. Myrberg Jr (1990) demonstrated that the noise produced by fishing vessels and their gear may cause fish to avoid these sources, potentially affecting the efficiency of certain fishing methods or vessel types. However, this remains a topic of debate, as the extent of avoidance behaviour appears to vary and is not consistently observed across studies (Fernandes et al., 2000). Thus, while it is evident that fish detect and respond to anthropogenic noise, it remains unclear whether they will move away, as responses likely vary by species, age, sound level, and other factors (Popper et al., 2003; Popper and Hawkins, 2021).

A second factor of interest due to its potential to affect the welfare of Atlantic salmon and other salmonids is artificial light. The light regime in commercial salmon farms is dominated by natural sunlight, particularly during the summer months. Artificial lights are used in these pens as management tools to, e.g., stimulate growth, suppress sexual maturation and impair/stimulate smoltification (Endal et al., 2000; Hansen et al., 2017) and are especially important in the darker months. Continuous artificial lighting is thus routinely superimposed on natural light during winter and spring in salmon farms (Oppedal et al., 1997). Light conditions in pens can also be influenced on shorter time scales by the light emitted by submerged equipment such as ROVs. Artificial light in pens has been found to perturb the spatial distribution of fish (Juell et al., 2003; Nightingale et al., 2006; Marchesan et al., 2005; Oppedal et al., 2007). For instance, Juell and Fosseidengen (2004) found that salmon rapidly adjusted their depth according to lamp position, suggesting that controlled light gradients can help reduce crowding and steer the fish away from areas where conditions are sub-optimal. Oppedal et al. (2001) observed that post-smolt salmon exposed to continuous artificial light in winter swam steadily at deeper, warmer layers, while those under natural light moved upward and became more dispersed at night. In spring, higher light intensity promoted fish to move upward earlier, and by summer, all fish groups preferred the warmer surface layers. Artificial light can also cause elevated stress levels in farmed salmon (Newman et al., 2015). In an experiment conducted by Migaud et al. (2007), post-smolt Atlantic salmon exposed to high-intensity blue LED light exhibited a temporary stress response characterised by elevated cortisol and glucose levels that normalised within a few hours. No such response was observed under white LED light or lower-intensity blue light. Additionally, no immune effects or retinal damage were observed in the experiment, suggesting that salmon can physiologically adapt to these lighting conditions. Earlier studies have found that light conditions can also have impacts on fish aggression (Valdimarsson and Metcalfe, 2001) in finding that fish stayed closer but were less aggressive when exposed to low light intensities, whereas the level of aggression increased as light intensity increased. These observations imply that while targeted underwater lighting may be used as an active measure to improve the welfare and health of farmed salmon, it is important to consider how the introduction of artificial lights may otherwise perturb fish behaviour and physiology.

Unmanned Underwater Vehicles (UUVs) are one example of equipment that introduces both acoustic noise and light into the pen environment. They are used at farms with increasing frequency, for example, in net inspection tasks, and thus have the potential to impact fish welfare at a relevant scale Kelasidi and Svendsen (2023). Consequently, their design in terms of noise and light emission should be motivated by knowledge on fish reactions to these factors. To address these knowledge gaps, our study explored how anthropogenic sounds and light variations affect the behaviour of farmed Atlantic salmon. We exposed Atlantic salmon at an industrial-scale fish farm to controlled sound emissions and artificial lights. Using acoustic (360 ° ) sonars, we quantified the fish’s response based on distance of the fish to the source before, during and after the stimuli. The data was analysed using an extension of the deep learning (DL) based approach for processing sonar data proposed by Zhang et al. (2024). The outcomes of the study contribute to quantifying and understanding the impacts of anthropogenic sounds and light changes on fish behaviour. This knowledge can in turn be used to derive regulations and specifications on how vessels, UUVs and new technologies should be designed and adapted for the aquaculture industry to improve fish welfare. The analysis method developed in this study is relevant for further studies into how different aquaculture systems and practices may affect farmed fish, and as a method for designing future tools that are less disturbing for the fish.

## 2 Materials and methods

### 2.1 Experimental setup and data acquisition

**FIGURE 1:**
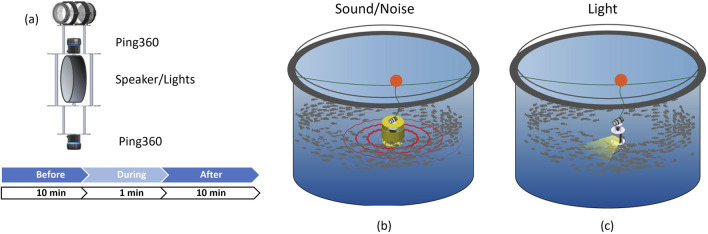
Experimental setup. The centre structure **(a)** was equipped with a Ping360 sonar on top and bottom. An underwater speaker **(b)** or light **(c)** was installed in the centre structure to stimulate the fish through acoustic or optical means.

**FIGURE 2:**
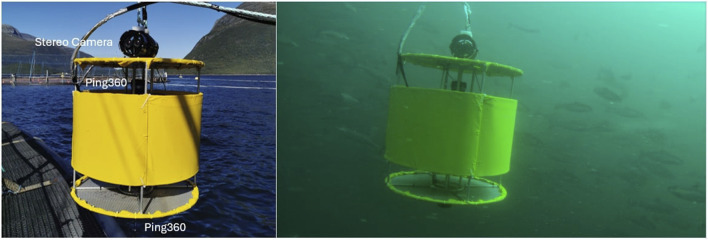
Centre structure setup for studying the impacts of sound (P1 and P2).

**FIGURE 3:**
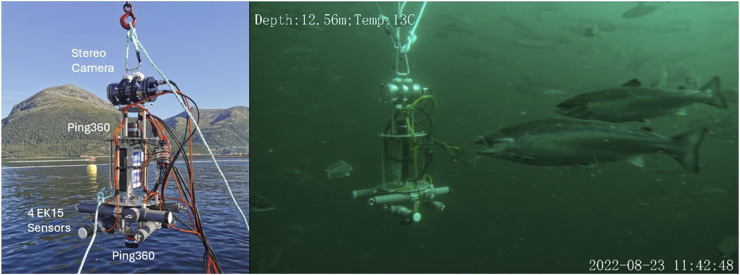
Centre structure setup for studying the impacts of varying light intensities (P3).

Three experimental field campaigns (P1, P2 and P3) were conducted in two salmon farms exposing fish of different sizes to different sound frequencies and light intensities (see Table 1 for an overview). The tests were conducted in industry scale net pens at SINTEF ACE (SINTEF, 2023) October 19–20 2021 (P1), August 08–09 2022 (P2), and August 23–26 2022 (P3), under calm conditions to avoid the influence of strong currents and waves, and during normal daylight hours (09:00–17:00) to minimise variability in natural light affecting fish perception. Feeding and other treatments were paused to eliminate potential confounding effects. All tests featured a cylindrical aluminium structure of 30 cm diameter and 60 cm height (referred to as the “centre structure”) that housed the sensors and actuators needed to both induce tailored sound and light disturbances and observe the resulting fish responses (Figure 1). 360° mechanical scanning image sonars (Ping360, BlueRobotics Inc.) were mounted on the top and bottom panel of the centre structure to enable observing the distribution of the fish around the centre structure with redundancy (Figures 2, 3).

**TABLE 1:** Overview of fish participating in field trials and factors tested.

Trial	Location	Pen	# Fish	Fish avg. Weight [kg]	Centre structure	Depth [m]	Date	Impact factors	# Repetitions
P1	Tristeinen	A	27,332	5.862	Big yellow cylinder	8	19 Oct 2021	Sounds: 100Hz, 200Hz, 400Hz, 600Hz and 1000 Hz	3
		B	99,243	4.989	Big yellow cylinder	8	20 Oct 2021	Sounds: 100Hz, 200Hz, 400Hz, 600Hz and 1000 Hz	3
P2	Korsneset II	C	194,536	0.962	Big yellow cylinder	8	08 Aug 2022	Sounds: 200Hz and 600 Hz	6
		D	193,476	0.961	Big yellow cylinder	8	09 Aug 2022	Sounds: 200Hz and 600 Hz	6
P3	Korsneset II	D	193,374	1.203	Center Structure	12	23 + 26 Aug 2022	Lights: Low, Medium, High, Very high	6
		D	193,374	1.203	Center Structure	8	24 Aug 2022	Lights: Low, Medium, High, Very high	6
		C	193,394	1.214	Center Structure	8	25 Aug 2022	Lights: Low, Medium, High, Very high	6

P1 and P2 were designed to study fish reactions to acoustic signals, and thus featured an underwater speaker (DRS-8 in P1 and DRS-8 MOD 2 in P2, Oceanears Inc.) installed inside the centre structure. The centre structure was then mounted inside a cylindrical yellow shell structure with dimension \diameter 60×60 cm that was suspended 8 m below the surface in both tests. This depth corresponds to a biologically active zone for fish during daylight hours (Føre et al., 2018), aligns with typical ROV operating depths in net pens, and falls within the waterproofing limitations of the equipment (Zhang et al., 2024). The underwater speaker was connected to a custom built amplifier placed topside that was controlled by mobile phone via Bluetooth, using the Android-application Tone Generator (TMSoft). This system was set to induce sounds at specific frequencies at set intervals using a high sound level (estimated at 180 dB re 1 µPa at 1 m) to maximize the chance that the fish would perceive the sound. Since farmed salmon hearing is more sensitive to low frequencies of approx. 100–580 Hz (Hawkins and Johnstone, 1978), and the acoustic noise measured in fish farms tends to be dominated by low frequencies (<1,000 Hz) (Radford and Slater, 2019; Barrett and Oppedal, 2025), the sound signals used in the trials spanned from 100 to 1,000 Hz. In P1, tones with centre frequencies 100 Hz, 200 Hz, 400 Hz, 600 Hz and 1,000 Hz were played in random order, each frequency occurring in total three times, while P2 featured only 200 and 600 Hz tones that were played sequentially six times. Each tone was maintained for 60 s, and there was a 10 min break between consecutive signals. The experiment was conducted in two different pens in both trials (Pens A and B in P1 and Pens C and D in P2), testing each sound frequency with 3 or 6 replicates across different pens.

In P3 the aim was to study fish’s reaction to light. Two SeaLED 300 LED lights (Imenco Inc.) were therefore installed on the centre structure (Figure 3), each of which could be set at three settings: 0 (=lights off), low, and high intensity. Combining the two lights resulted in four distinct light settings: low = 600 lx (one light off, one light on low), medium = 1,100 lx (both lights on low), high = 6,600 lx (one light on low, one light on high), very high = 14,500 lx (both lights on high). Since the LED lights needed to be visible for the fish, the centre structure was not clad in the cylindrical shell structure during P3. This experiment was also conducted in two pens to achieve fish group replication (Pens C and D). Additionally, since natural light strongly affects the light conditions in the pen, the trial was repeated at a deeper depth (12 m) in one pen (Pen D) to see if fish responses differed when natural light levels were lower. In the tests conducted at 8 m depth, each of the four light intensity levels was turned on six times at random, each treatment lasting 60 s, and with a 10 min break between samples (as for sound inP1 and P2). The 12 m depth trial was similarly set up, but with a total of six repetitions in one single pen divided between 2 days (four on August 23 + two on 26 Aug 2022).

### 2.2 Data analysis methods

This section describes the DL approach used to process the acoustic data and the statistical methods used to study fish behaviour changes.

#### 2.2.1 Sonar data processing

**FIGURE 4:**
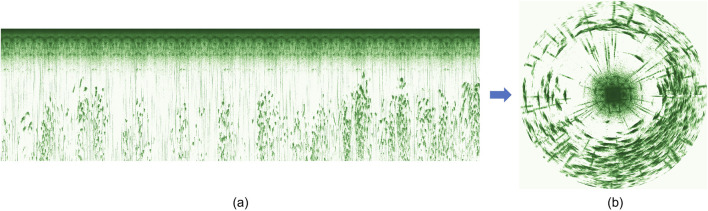
Sonar data representation showing **(a)** the raw output from the sonar and **(b)** the transformed circular scan representation.

The Ping360 sonar was selected for this study because it offers a full 360-degree field of view and performs reliably in turbid or low-light conditions where optical sensors may be ineffective, enabling comprehensive, wide-area monitoring of fish distribution around the sonar. Sonar data were processed using a DL-based method (Zhang et al., 2024) that automatically identified fish distribution patterns and estimated the average avoidance distances (in m) of salmon surrounding an intrusive object. This was done by first converting the sonar output, which consisted of time series of intensity values reflecting echo strength, from Cartesian to polar coordinates. The results of this conversion were circular-scan images that provide a 360-degree view of the sonar’s surroundings where fish and other objects are marked by higher intensity than empty volumes (Figure 4). This method was based on the assumption that salmon responses when facing intrusive objects would occur as annular distributions centred at the object in the sonar images.

The images were then subjected to a UNet++ model (Zhou et al., 2020), a DL semantic segmentation network with a symmetric U-shaped encoder-decoder architecture and nested skip connections. This model identified fish avoidance patterns by deriving the fish avoidance distance as the average of the distance from each pixel along the inner perimeter of the distribution to the centre of the object. Further details of the model and methodology can be found in Zhang et al. (2024).

**FIGURE 5:**
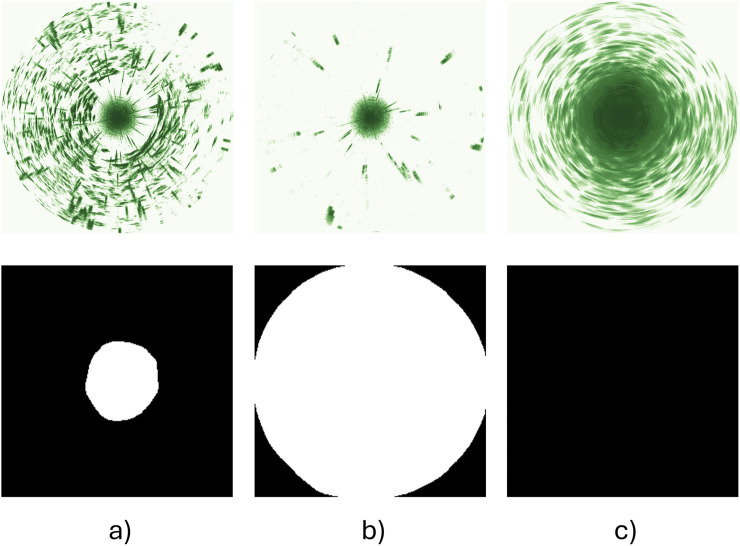
Three categories of training images (first row) and corresponding labels (second row). **(a)** Valid data where fish swimming pattern is clear, labelled by a solid white polygon; **(b)** Valid data where fish are out of sonar range, labelled by a solid white disk covering the sonar scan area; **(c)** Invalid data where fish pattern is unclear or noisy, labelled by a black mask.

Although Zhang et al. (2024) successfully applied this method to study the avoidance distance of fish to structures of different shapes, sizes and colours, they only classified sonar images into two categories: valid data where clear fish distribution patterns could be identified (denoted as solid white polygons covering the areas devoid of fish, Figure 5a), and invalid data where patterns could not be identified (denoted as black masks, Figure 5c). While these categories were sufficient to distinguish stationary responses toward different structure types, the present study was expected to elicit more acute responses such as flight responses when exposed to abrupt changes in sound or light. This type of response could result in data where all fish were out of sonar range, which would be of high interest in the study, but that would be classified as invalid data according to the original categories. An additional output category was therefore added where a solid white disk covering the sonar scan area was used to indicate the absence of fish (Figure 5b). The DL model was retrained on a new dataset containing all three categories (i.e., valid data with clear fish distribution patterns, valid data with fish outside sonar range, and invalid data where fish distributions are unclear or noisy, Figure 5).

**FIGURE 6:**
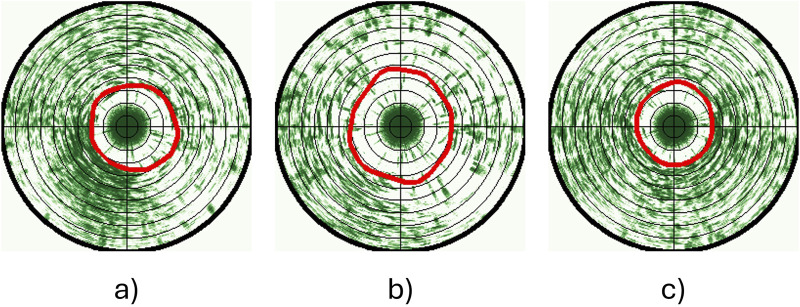
50-s CFP images and detected fish swimming patterns (red curves) **(a)** before, **(b)** during, and **(c)** after 400 Hz sound exposure. Concentric rings indicate steps of 1 m distance.

We only used data from the bottom sonar for analyses in this study since there were fewer obstructions (e.g., cables, camera) in its observation volume than around the top sonar. This does not bias the data, as previous analyses (Zhang et al., 2024), along with assessments using similar pen systems and the trial data in this study, showed that the top and bottom sonars consistently gave comparable outputs when there are no obstructions. Circular scans acquired over 1 min before, 1 min during and 1 min after exposure to sound/light signals (i.e., 3 min in total) were analysed to study the behaviour changes of fish in response to the stimulation. When deriving the Cumulative Fish Presence (CFP) images used to analyse fish group responses, the first and last 5 s that coincide with transition periods between treatments were discarded to avoid impacts from the previous state or inaccurate timing. In sum, each replicate CFP image represents sonar data for 50 s before, 50 s during, and 50 s after the treatment. Once the CFP images had been categorised and a greyscale image similar to those presented in Figure 5 had been acquired, the contour of the white region was identified. The fish avoidance distance for each dataset was then found as the average distance to the pixels comprising the resulting contour, enabling a comparison of fish responses before, during and after exposure to a treatment (see example output in Figure 6).

In addition to CFP images, single-circular-scan images, each of which represents fish distribution captured during a circular/cycle scan (approx. 8 s), were processed to analyse fish behavioural changes during treatment transition periods. These results are presented as Supplementary Material. Over 378 50-s CFP images and 3,028 one-circular-scan images were automatically processed using the updated DL model. Of these, only 95 single-circular-scan images required manual correction due to inaccuracies, demonstrating the effectiveness of the updated model.

#### 2.2.2 Statistical method

Data from the 50 s CFP image triplets (before/during/after) were used to analyse effects of sound and light on the distance of the fish to the source. Permutational Analysis of Variance (PERMANOVA, Primer v.7) was employed to compare distances measured before, during, and after exposure (factor ‘Timing’, 3 levels, fixed). Moreover, the analyses included the different frequencies or light intensities (factor ‘Frequency’, 5 levels in P1 and 2 levels in P2; or ‘Light Intensity’, four levels, in P3, fixed) as well as the experimental pen (factor ‘Pen’, 2 levels, random), or the water depth (factor ‘depth’, 2 levels, fixed) for the data from Pen D in P3. For a detailed overview including number of replicates, see Table 1 and Supplementary Table S1 (in Supplementary Material). Comparisons were based on Euclidean distance and 9999 unrestricted permutations of residuals under a reduced model with a significance level of 5%. Where the number of unique permutations was < 100, the Monte Carlo asymptotic pMC-value was consulted. Where PERMANOVA indicated no significant differences between factors (significance level ≥ 25%), the term was pooled to increase power (Anderson, 2017; Anderson et al., 2008).

## 3 Results

### 3.1 Effect of sounds

**FIGURE 7:**
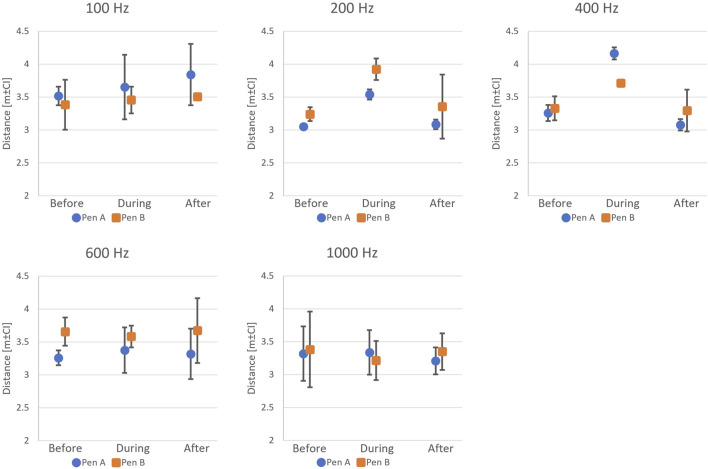
Fish avoidance distance estimates before, during, and after sound exposure in P1 (n = 3 replicates per frequency). A clear avoidance response was observed at 400 Hz (During > Before = After), while other frequencies (100, 200, 600, 1,000 Hz) showed no significant response.

The 50-s CFP data from P1 shows that salmon in Pens A and B reacted to 400 Hz sound by increasing the distance to the sound source on average by 28% and 12%, respectively. This indicates avoidance behaviour. Notably, when the sound stopped playing, distance between the fish group and the structure returned to the original distance (Frequency x Timing, ${\text{F}}_{8,89}$ = 2.131; p = 0.045, followed by pairwise comparisons with p < 0.05 for During > Before = After; Figure 7c). This redistribution pattern suggests a temporary displacement of fish away from the structure during sound exposure, followed by reaggregation once thedisturbance ended. Sounds played at 100, 200, 600 or 1,000 Hz did not cause any significant change in the distance the salmon kept to the structure. This suggests that the fish in this study were particularly sensitive to 400 Hz, possibly because of the overlap between 400 Hz and species-specific auditory sensitivity or structural resonance frequencies.

**FIGURE 8:**
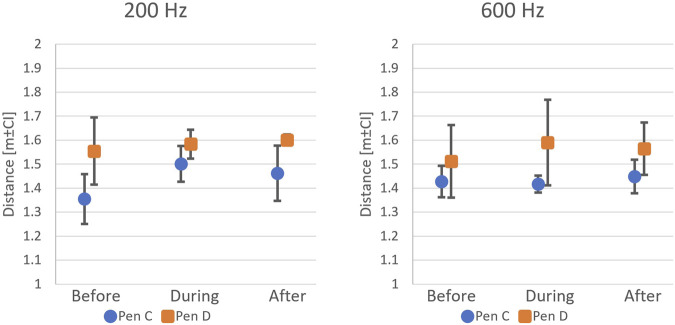
Fish avoidance distance estimates before, during, and after sound exposure in P2 (n = 6 replicates per frequency). No consistent avoidance pattern was observed at either 200 Hz or 600 Hz.

Similar results were observed in P2, with no effect of the onset of sound on the distance of salmon from the sound source measured for 200 or 600 Hz. Here, the only differences were found between the two test pens, where fish generally stayed further away from the structure in Pen D than in Pen C (Pen, ${\text{F}}_{1,71}$ = 30.27; p < 0.001; Figure 8). This spatial discrepancy likely reflects environmental or behavioural differences between pens rather than sound exposure effects.

### 3.2 Effect of light

**FIGURE 9:**
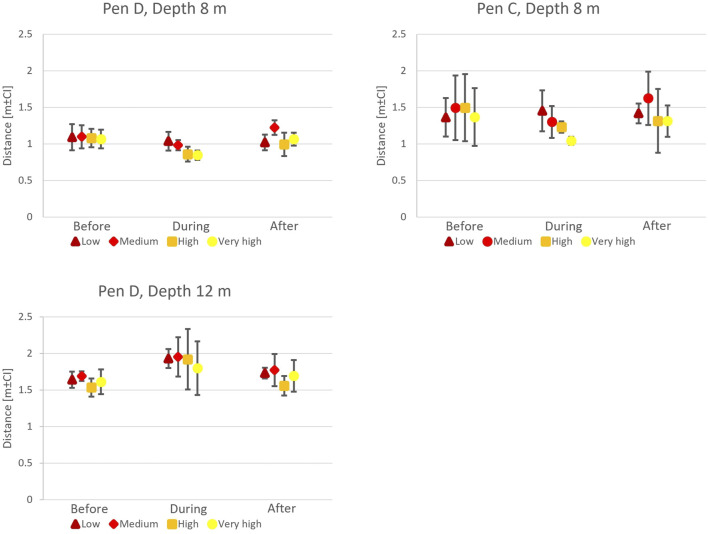
Fish avoidance distance estimates before, during, and after light exposure in P3. At 8 m depth, fish moved closer to the light (During < Before = After) for medium, high, and very high light intensities. At 12 m depth, fish moved away from the light (During > Before = After) for all light intensities.

When exposed to artificial light at a depth of 8 m, 50-s CFP data showed that the salmon swam closer to the light source when light was turned on (Timing, ${\text{F}}_{2,143}$ = 7.886; p < 0.001), demonstrating an attraction response. Moreover, there was a trend towards an increasing effect with increasing light intensity (Light Intensity, ${\text{F}}_{3,143}$ = 3.888; p = 0.011, Figure 9). That is, fish redistributed more tightly around the structure at higher intensities, suggesting a intensity-dependent behavioural response. This applied to both Pens C and D, though the salmon in Pen C generally kept a larger distance than fish in Pen D (Pen, ${\text{F}}_{1,143}$ = 78.753; p < 0.001).

Additional tests conducted at 12 m depth in Pen D showed a contrasting reaction where fish moved away from the light source, independent of the light intensity (Timing x Depth, ${\text{F}}_{2,143}$ = 21.899; p < 0.001). Moreover, fish generally kept a larger distance to the test structure in 12 m depth than in 8 m depth, suggesting a depth-related shift in their behavioural response to artificial light.

## 4 Discussion

We conducted three field trial campaigns to investigate the effects of artificial sound and light on the behaviour of Atlantic salmon (*Salmo salar* L.). A DL-based method was used to analyse sonar data from 50 s before, during, and after sound/light exposure, enabling us to assess fish responses to these stimuli.

In both sound and light experiments, the salmon showed selective responses to external stimuli, reacting differently based on the intensity, frequency, or environmental context. The fish exhibited an escape response when exposed to sound at 400 Hz, but demonstrated no clear reactions to sound at the other frequencies. Responses toward the introduction of artificial light were varied, with the salmon swimming closer to the light source when tested at 8 m depth and conversely moving away from the source when tested at 12 m depth. However, in sum, these findings illustrate that the introduction of specific acoustic noises or artificial lights in net pens may have an impact on the distribution and behaviour of farmed fish. In turn, this may also induce less favourable conditions such as reduced welfare and stress, meaning that care should be taken when designing and planning pen operations using technological tools that may emit light and sound.

### 4.1 Responses to sounds

The salmon exhibited a noticeable reaction when exposed to sounds at 400 Hz, where they abruptly moved away from the sound source^1^, demonstrating that the fish were able to sense the acoustic pulse, either auditory or through the lateral line organ. This behaviour was distinct and consistent across pens, suggesting a heightened sensitivity of fish to this particular frequency, which is consistent with previous studies reporting that Atlantic salmon have a relatively narrow hearing bandwidth up to approx. 300–500 Hz (Popper and Hawkins, 2019). The response resembles escape behaviour, which may indicate that the fish were startled by the sudden onset of the sound signal. Since the fish group structure settled back to the original distance after the sound stopped, it is likely that the response was directly linked to the acute sound stimulus. This resembled escape responses toward sound signals that have previously been observed in salmonids (Knudsen et al., 1997; Bui et al., 2013; Oppedal et al., 2025). While this implies that the findings in the study were in consensus with earlier findings, it should be noted that the previous studies used lower frequencies (1–150 Hz), and observed escape behaviours mostly when exposing the fish to signals in the infrasound range ( ≤ 12.5 Hz) (e.g., Oppedal et al., 2025).

The fish did not show a clear response when exposed to the acute emission of sounds at other frequencies (i.e., 100 Hz, 200 Hz, 600 Hz and 1,000 Hz). This lack of reaction may indicate that sounds at these frequencies were not perceived as an uncomfortable disturbance or as an indication of some increased risk by the fish. An alternate explanation for the lack of response for these frequencies can be that the sounds were outside the primary hearing range of the fish. While, according to Hawkins and Johnstone (1978), 100, 200 and possibly 600 Hz should be inside or close to the normal hearing range of salmon, the hearing loss caused by otolith deformities observed in farmed salmon (Reimer et al., 2016) could vary with frequency. If so, the fish may have been unable to detect the sounds with sufficiently clarity to distinguish them from the ambient environment, and hence not have any motivation to respond. This is supported by earlier studies that found that Chinook salmon with hearing loss in the 100–300 Hz range due to otolith deformities also showed a loss in hearing sensitivity for adjacent frequencies including 400 Hz (Oxman et al., 2007). Although Chinook salmon and Atlantic salmon are not the same species, this implies that hearing loss due to otolith deformation is likely to impact the whole aural range and not only a few selected frequencies.

In this context the lack of a clear reaction to sounds at 200 Hz was unexpected as previous research had indicated higher sensitivity for 100–300 Hz than for 400 Hz (Oxman et al., 2007). This discrepancy might reflect interspecies variation, differences in experimental design, or variability in hearing ability due to aquaculture-related otolith deformities. Another element to consider in this discussion is that the acoustic power level used in this study (estimated at 180 dB re 1 µPa at 1 m) was higher than that observed in studies exploring soundscapes at fish farms (which typically range from 98 to 112 dB re 1 µPa at 1 m, Radford and Slater, 2019; Barrett and Oppedal, 2025). This means that the sound signals played during the trials were louder than the ambient noise level at the farm and implies that the fish should have been able to perceive these signals clearly, yet a response was only observed at 400 Hz. More specific research into the aural properties of Atlantic salmon is needed to conclude whether the absence of response toward sounds with frequencies other than 400 Hz is due to hearing impairments or the fish making a conscious decision of not responding.

Further studies into the hearing of farmed salmon could help shed light on these aspects.

### 4.2 Responses to light

The salmon consistently stayed closer to the light when it was placed at 8 m, an effect that was modified by light intensity as the fish moved closer to the source when the intensity was increased. This suggests that the fish were attracted to the light at 8 m depth but appeared to avoid it at 12 m depth. Both these patterns were emphasised when light intensity was increased (see Supplementary Figure S3, Supplementary Material). These findings reflect observations by Juell et al. (2003) and Juell and Fosseidengen (2004) where fish tended to cluster more tightly around lights on the surface or in shallow water but became more dispersed when lights were placed deeper. The impact of artificial lights on the spatial distribution and behaviour of fish within the water column is thus a more complex combination effect of light intensity and depth rather than solely depending on light intensity.

Closer to the surface, the impact of natural light on the pen environment is stronger, meaning that the introduction of artificial lights at shallow depths may not affect lighting conditions as much as it will deeper in the pen. Moreover, the fish population in the pen may be stratified, with those seeking surface light, e.g., to feed, being present in the upper meters and showing positive phototaxis. In contrast, fish in lower depths may be actively avoiding the daylight on the surface, and thus may also show negative phototaxis and avoidance to the artificial light, as seen in the experiment at 12 m depth. Such variation in phototaxis has been described in other studies (Oppedal et al., 2011) and may vary throughout the population in the pen. For example, Wright et al. (2015) used light at night to attract fish to a specific depth but did not find the reaction to be consistent for the entire population in the pen, with some fish following the light while others showed no reaction.

### 4.3 Experimental design and stability of pen environment

This study utilised a hierarchical replication approach: (a) within-pen replication, involving repeated testing of each impact factor under consistent conditions within the same pen to isolate factor effects; and (b) between-pen replication, involving the repetition of identical trials across different pens at different time to capture natural environmental variability typical of aquaculture systems. This dual-scale approach enabled distinguishing the tested impact factor effects from potential confounding factors associated with spatio-temporal environmental variations, thus enhancing the robustness and validity of our findings. Previous research has shown that strong currents and large waves can influence fish swimming performance and behaviour, particularly in smaller individuals with lower swimming capacity. However, such effects are generally associated with prolonged exposure. Short-term experiments (on the scale of hours), as in our study, are unlikely to be significantly affected by variations in fish swimming behaviour due to environmental conditions (Hvas et al., 2021; Zhang et al., 2024).

Each 60 s stimulation in the trials was followed by a 10 min break period that allowed the fish to return to baseline behaviour, and also effectively provided a “no-disturbance” reference condition within the same environment. To control for potential behavioural drift or disturbance effects, we assessed the behaviour of the fish across three distinct phases: before, during, and after the stimulus. There was no statistical difference between before and after the fish were exposed to sound or light. As the entire school of fish tends to move in a circular pattern, the fish being exposed to a stimuli at a certain time and potentially reacting to it are unlikely to stay in the area after responding. Thus, fish in the observation area after the stimuli was switched off, were probably unstimulated fish that had not responded to the original treatment but are entering the area for the first time. Moreover, the similarity in avoidance distance before and after exposure suggests that the pen environment remained stable during the experiments and the experiment only affected fish in immediate vicinity to the structure. In sum, this indicates that the responses exhibited by the fish were primarily elicited by the intended sound/light stimuli and not due to other factors (e.g., long-term habituation, environmental variability, or procedural disturbances such as external disruption clearing entire pen areas), supporting the validity of our experimental design and data collection.

Finally, the observed fish distribution patterns are also consistent with the hypothesis that fish respond to stationary stimuli placed within the pens by spatially distributing in a circular pattern centred around the source of the disturbance. Such circular avoidance patterns likely reflect a natural schooling response to internal localised perturbations. In contrast, external disturbances, such as predators or maritime traffic, would likely elicit different spatial responses, potentially in the form of directional rather than symmetrical avoidance patterns. Future studies could explore how external disturbance directionality influences the shape and extent of avoidance behaviour, providing deeper insight into the complexity of fish responses under varying impact factors.

### 4.4 DL-based sonar data analysis method

In this study, we applied a DL-based semantic segmentation approach to sonar data to analyse fish avoidance distance in response to sound and light. Conventional sonar processing techniques, such as threshold-based segmentation, usually rely on raw signal intensity to detect objects present in the data. These methods often capture not only fish but also the reflections from other objects including central structures, the sonar itself or other devices present in the water volume. Because of this, estimating true fish avoidance distances using such approaches will require additional post-processing steps, which in turn reduces the autonomy of the method and may introduce new errors and inaccuracies in the outcomes. The DL-based method in contrast leverages the capacity of convolutional neural networks (CNNs) to learn complex spatial patterns and contextual features embedded in the sonar data, and directly identifies the specific regions where fish avoid the sound/light. This enables a more direct, robust, and targeted quantification of avoidance behaviour, while minimising the influence of background noise and structural artifacts. Although the original method as presented by Zhang et al. (2024) was found to provide reliable results, the introduction of the second class of invalid data in the present study further improved the prediction accuracy of the method.

### 4.5 Main implications and applications

Our results provide valuable information on fish reactions to sound and light in net pens. This data can be used to design underwater vehicles, machinery, and other sound-emitting human systems/activities to operate at frequencies that have less chance of disturbing farmed fish. For instance, Cai and Bingham (2011) found that the motors of a standard ROV emitted noise at 400–500 Hz, which overlaps with the frequency found to elicit an avoidance response in the salmon in the present study. Designing new vehicles equipped with motors that emit sounds with frequencies outside this range could thus reduce the risk of startling or displacing fish populations during ROV operations. However, care must then be taken to also avoid the lower frequencies that have previously been identified to have adverse effects on farmed fish ( ≤ 100 Hz, Knudsen et al., 1997; Bui et al., 2013). Such design measures could also help minimising the environmental impact of vehicle activities on aquatic ecosystems in general, as it is likely that wild fish have similar sensitivities to sounds as farmed salmon.

At the same time, this apparent sensitivity to 400 Hz could also be exploited in developing more effective fish management strategies. Emitting sounds that repel the fish could, for example, be employed to guide fish movement in aquaculture systems. Underwater speakers could thus be used to deter fish away from areas where they may be in danger, e.g., near inlets/outlets or other equipment. Alternatively, this approach could also be used to steer the fish into specific areas to facilitate easier capture or handling, possibly reducing the need for handling and the use of crowding nets. In this context, the potential of the fish habituating to an originally repellent sound frequency may need to be assessed.

The findings on responses toward artificial lights could likewise be used in the development of new technological tools. Developing “fish-friendly” light sources that are less likely to have negative impacts on fish behaviour, physiology, and ecosystems is a particularly promising direction in this area. Underwater vehicles or exploration systems could, for example, be set up to use dimmer or more diffuse lighting in deep waters than when closer to the surface to minimise their impact on the natural behaviour of fish. Moreover, turning up lights gradually when a mission commences may be more beneficial than simply turning all lights on as it may prevent the fish being startled. While such a reduction in light intensity would also reduce visibility for the cameras on the ROV, this can be countered by using more advanced cameras that can operate on lower light levels.

The expanded sonar based method for identifying fish distribution patterns developed in this study could also be used in other studies aiming to scope the responses of fish toward external stressors. While the present study focused on the effects of sound and light, there are several other acute or chronic factors known to have impacts on fish behaviour and distribution patterns (Oppedal et al., 2011). In cases where such factors are localised (i.e., can broadly be considered point sources), the same method based on sonar data and DL could shed light on their impacts on the fish. The method could also be further developed as a tool for continuous assessment of noise/light pollution at farming sites since it will only require the permanent deployment of a sonar attached to a topside computer for processing. This would be a simpler technical solution than the conventional approach of using stationary echo sounders as one Ping360 unit could cover a volume that would otherwise require several echo sounders. The resulting data could allow the correlation between sound/light levels and behavioural changes in the fish (e.g., feeding patterns, stress responses, and swimming activity). Such a tool could help the farmer adjust operations to reduce the elicited response, which may in turn lead to improved fish welfare during production.

## 5 Conclusion and future work

In this paper, we studied the avoidance distance of fish under the influence of different sounds and lights based on the data collected from 1-min short-term exposure experiments, revealing insights into how fish respond to both sound and light stimuli. Our results show that fish responded to sounds at 400 Hz by exhibiting avoidance behaviour, while 100, 200, 600, and 1,000 Hz did not elicit a response. Responses toward artificial lights depended on light intensity and water depth, with deployment at shallower depths inducing attraction toward increased light levels, and deeper deployments inducing avoidance.

An important future element in exploring this topic further will be to conduct experiments collecting data when fish are subjected to long-term stimulation by different aural and visual impact factors. This will provide more insights into the chronic response of the fish toward such signals, which will complement the results obtained in these trials describing acute response patters occurring when conditions change abruptly. Gaining such knowledge will be important to understand the impacts of continuous or long-lasting operations at farming sites such as feeding and vessel operations.

Another future measure could be to combine the measurement and analysis method used in the present study with methods based on stereo cameras. While the sonar solution is useful for gauging the distribution patterns, stereo cameras are better equipped to capture short term responses such as changes in swimming speed, movement trajectories or direction changes (Alvheim et al., 2024). The quantification of fish responses at this level of detail can further be used to extend existing numerical models of farmed fish behaviour (e.g., Føre et al., 2009) such that they can also consider fish interactions with underwater vehicles, thereby enabling the simulation of autonomous operations in fish farms.

An important final point to note with regards to this study, is that there are currently few official regulations addressing the impacts of sound and light on fish in aquaculture. However, due to the increased focus of both the public and authorities on animal welfare in aquaculture, such regulations are likely to emerge in the coming years. The development of robust, evidence-based standards is therefore necessary for promoting sustainable aquaculture practices and ensuring fish welfare during production. This requires defining acceptable noise levels and light intensities for different species and life stages. Our current and ongoing research studies will help develop these standards by providing data on specific thresholds that fish can tolerate without adverse effects.

## Data Availability

The original contributions presented in the study are included in the article/Supplementary Material, further inquiries can be directed to the corresponding author.

## Footnotes

1 This behaviour was also evident in the before-during-after curves for all 400 Hz sound tests shown in Supplementary Figure S1 (Supplementary Material), which exhibit a characteristic “ > ” shape, indicating an increase in distance during 400 Hz sound exposure, followed by a return to baseline levels once the stimulus ceased
