# Supplementary material for: Avoidance behaviours of farmed Atlantic salmon (Salmo salar L.) to artificial sound and light: a case study of net-pen mariculture in Norway
Source: Front Robot AI. 2025 Sep 11;12:1657567. doi: 10.3389/frobt.2025.1657567 (PMC12460098; doi:10.3389/frobt.2025.1657567)
Supplement: Supplementary file 1 [file DataSheet1.pdf]

Supplementary Material

1 SUPPLEMENTARY DATA

Data of each replicate experiment

Table S1. Fish responses related to different sounds and lights.

**Data**	**Factor**			**Effect**	**Pairwise comparison**
**P1**	Frequency	Fixed	100, 200, 400, 600, 1000	Frequency x Timing, $F_{8,89}$=2.131; p=0.045	At 400 Hz, fish are further away after onset of sound, no difference in distance between before and after when sound has stopped. No reaction to the other frequencies.
19+20 Oct 2021	Timing (Onset of sound)	Fixed	Before, During, After		
	Pen	Random	Pen A, Pen B		
**P2**	Frequency	Fixed	200, 600	Pen, $F_{1,71}$=30.27; p<0.001	No effect of onset of sound.<br>Fish in Pen D keep greater distance than fish in Pen C.
08+09 Aug 2022	Timing (Onset of sound)	Fixed	Before, During, After		
	Pen	Random	Pen C, Pen D		
**P3**	Light intensity	Fixed	1, 2, 3, 4	Timing, $F_{2,143}$=7.886; p<0.001	Fish come closer when the lights are turned on.
24+25 Aug 2022	Timing (Onset of sound)	Fixed	Before, During, After	Light Intensity, $F_{3,143}$=3.888; p=0.011	Trend: The effect increases with light intensity.
data from 8 m depth only	Pen	Random	Pen C, Pen D	Pen, $F_{1,143}$=78.753; p<0.001	Fish in Pen C keep greater distance than fish in Pen D.
**P3**	Light intensity	Fixed	1, 2, 3, 4	Timing x Depth, $F_{2,143}$=21.899; p<0.001	Independent of light intensity:
23+24+26 Aug 2022	Timing (Onset of sound)	Fixed	Before, During, After		Timing: Onset of light causes fish to distance themselves at 12 m, and to come closer at 8m.
data from Pen D only	Depth	Fixed	8 m, 12 m		Depth: fish at 12 m depth keep greater distance than at 8 m.

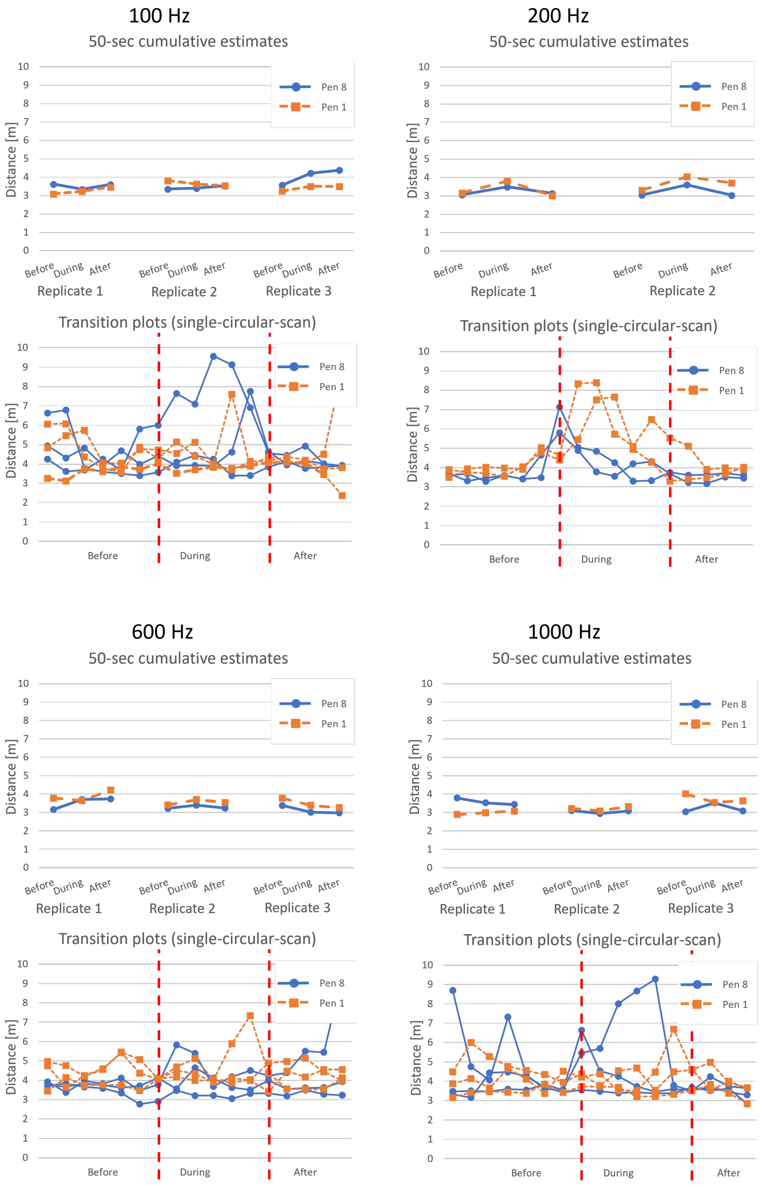

Figure S1: Fish avoidance distance estimates before, during, and after sound exposure in 2021 (individual replicates). At 400 Hz, fish showed increased avoidance distance during exposure, returning to baseline afterward. No consistent pattern was observed at other frequencies.

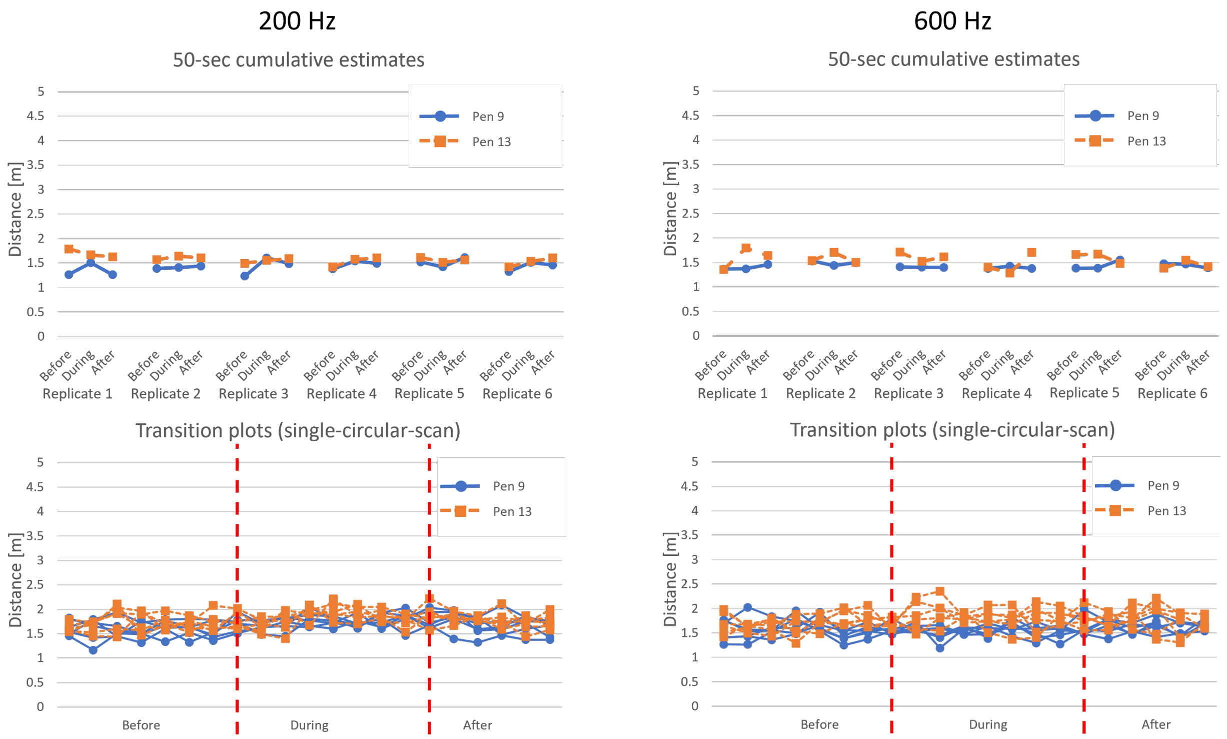

Figure S2: Fish avoidance distance estimates before, during, and after 200 and 600 Hz sound exposure in 2022 (individual replicates). No significant changes in avoidance distance were observed across exposure periods for either frequency.

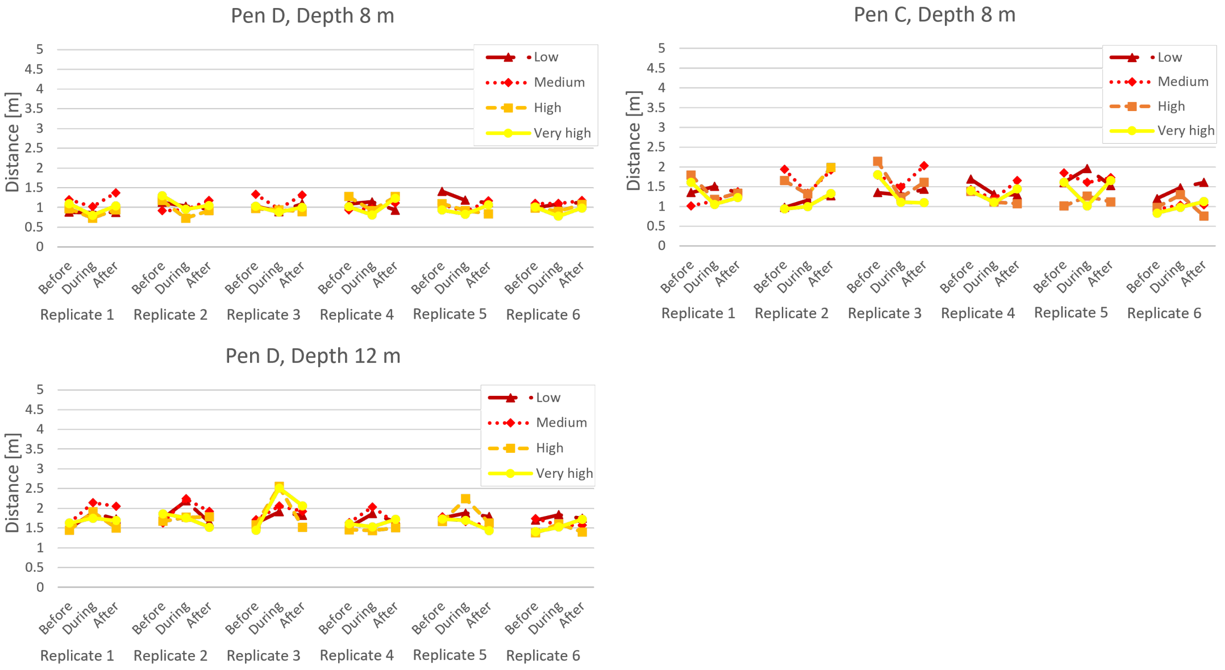

Figure S3: Fish avoidance distance estimates before, during, and after light exposure in 2022 (individual replicates). At 8 m depth, fish generally moved closer to the light during exposure (During $<$ Before = After) under medium to very high intensities. At 12 m, fish tended to move away (During $>$ Before = After) across all intensities.
